# Evaluation of the genomic alterations in the androgen receptor gene during treatment with high-dose testosterone for metastatic castrate-resistant prostate cancer

**DOI:** 10.18632/oncotarget.27408

**Published:** 2020-01-07

**Authors:** Marcus Moses, Ulkuhan Koksal, Elisa Ledet, Charlotte Manogue, Patrick Cotogno, Brian Lewis, Jodi Layton, A. Oliver Sartor, Pedro Barata

**Affiliations:** ^1^Tulane Cancer Center, School of Medicine, Tulane University, New Orleans, LA, USA

**Keywords:** castration-resistant prostate cancer, high-dose testosterone, next-generation sequencing, androgen receptor

## Abstract

Introduction: Castration resistant prostate cancer (CRPC) has been characterized by a reactivation of the androgen receptor (AR) signaling pathway via alterations in androgen metabolism and AR aberrations. High-dose testosterone (HDT) is emerging as an active treatment in metastatic CRPC, however, biomarkers of response are unknown. We hypothesized that responses to HDT might impact the genomic expression of AR alterations found in circulating-tumor DNA (ctDNA).

Methods: Retrospective analysis of mCRPC patients treated with HDT (testosterone cypionate q 2–4 weeks) with available clinical and somatic genomic data using a commercially available assay (Guardant360, Redwood City, CA). Clinical outcomes included PSA response (PSA50), time to PSA progression (TPP) and safety.

Results: A total of 33 mCRPC patients were treated with ≥2 testosterone cypionate injections. ctDNA testing revealed alterations in AR (39%), TP53 (48%), and DNA repair genes (12%). HDT was given for median of 4.0 months (95% CI, 2.6–5.3) with 24% of PSA50. Twenty patients were re-challenged with abiraterone (*n* = 2) or enzalutamide (*n* = 18) with 30% PSA50. Significant (grade ≥3) adverse events were observed in 5% of patients (grade 4 thrombocytopenia and asthenia). Patients with median baseline ctDNA% of ≥1.10 had numerically worse TPP outcomes and all patients with AR alterations exhibited decreased AR expression post-HDT (*n* = 9), yet no association between clinical outcomes and ctDNA findings was observed.

Conclusions: HDT led to a decrease in AR copy number and mutations which was independent from responses to therapy. Further understanding of the genomic alterations as potential predictor of response to HDT is needed.

## INTRODUCTION

Metastatic castrate resistant prostate cancer (mCRPC) remains a significant challenge for clinicians today. Among FDA-approved mCRPC therapies, novel hormonal therapies like abiraterone and enzalutamide, improve overall survival and provide initial clinical responses by targeting the androgen receptor (AR) pathway, but disease invariably progresses and further responses to AR pathway-directed therapy are in most cases, limited [1–3].

Castration-resistant disease has been characterized as dependent upon reactivation of the AR signaling pathway via alterations in androgen metabolism and AR gene aberrations, including alterations such as point mutations, gene amplifications, and ligand-independent splice variants [4, 5]. Adaptive overexpression of the AR and AR ligands promotes androgen-dependent growth resisting the castrate microenvironment in mCRPC [6]. Identification of these genetic biomarkers via next-generation sequencing (NGS) provides a better understanding of treatment resistance in mCRPC [7–9].

In the context of chronic testosterone suppression and despite tumor resistance to conventional antiandrogen therapy, higher doses of testosterone may have a positive therapeutic effect in a subset of mCRPC patients [10, 11]. Several studies have investigated the use of high-dose testosterone therapy (HDT) within castrate-resistant prostate cancer, either continuously using transdermal patches/gels or intermittently with intramuscular injections [12–16]. Although continuous and intermittent administration of exogenous testosterone are both viable strategies, more data is available with intermittent injections, coined “bipolar androgen therapy” (BAT) [10]. Ongoing Phase II trials (RESTORE & TRANSFORMER), have demonstrated successful administration of HDT via BAT resulting in clinical responses, low toxicity, improved quality of life, and re-sensitization to enzalutamide; thus justifying HDT as an emerging novel alternative therapeutic approach [16].

While there is preliminary clinical data supporting HDT as a therapeutic option, biomarkers of response are unknown [16]. In this pilot study, we assessed the safety and efficacy of HDT in mCRPC and analyzed the impact of genomic changes in the AR gene in the clinical outcomes of these patients.

## RESULTS

### Baseline clinical characteristics

Between May 2016 and April 2019, a total of 33 mCRPC patients, median age of 73 (60–88), 88% Caucasian and a median initial PSA 29.3 ng/mL (0.04–845), were treated with HDT. The majority (61%) had bone only disease, 36% lymph node, and 3% with visceral metastases. Prior to HDT, patients received a median of 2 (1–10) lines of treatment for CRPC (multiple lines of the same therapy were included), including second generation hormonal therapies (100%), taxanes (33%), immunotherapy (Sipuleucel-T, *n* = 8; Pembrolizumab, *n* = 2) (24%), and radium-223 (24%). Most patients (88%) received abiraterone (*n* = 16), enzalutamide (*n* = 7), or both sequentially (*n* = 6) immediately prior to HDT administration for a median 11.2 months (95% CI, 6.4–15.9). Additional baseline patient clinical characteristics are summarized in Table 1.

**Table 1:** Baseline characteristics prior to high-dose testosterone patients (*n* = 33)

**Race**	*n* (%)
Caucasian	29 (88)
African-American	3 (9)
Other	1 (3)
**Median Age**	*n* (range)
Treatment	73 (60–88)
**ECOG PS**	*n* (%)
0–1	24 (73)
2	1 (3)
Unknown	8 (24)
**Gleason Score**	*n* (%)
6–7	16 (48)
8–10	15 (46)
Unknown	2 (6)
**Metastatic Disease**	*n* (%)
Bone Only	20 (61)
Lymph Node	12 (36)
Soft Tissue	1 (3)
**Lab Values**	*n* (range)
PSA	29.3 (0.04–845)
Hemoglobin	12 (8.3–14.7)
ALP	99 (37–541)
LDH	196 (91–871)
Baseline Nadir Testosterone	272 (60–1374)
**Previous CRPC Therapies**	
Median	2 (1–10)
Abiraterone	25 (76)
Enzalutamide	21 (64)
Radium-223	8 (24)
Taxanes	11 (33)
Immunotherapy	8 (24)
**Oral Antiandrogen Treatment Prior to HDT**	*n* (%)
Abiraterone	16 (48)
Enzalutamide	7 (21)
Sequential Abi and Enza	6 (18)

### HDT and oral antiandrogen therapy re-challenge

Thirty-three patients received testosterone cypionate injections q 2–4 weeks (w) (2w, *n* = 6; 3w, *n* = 13; 4w, *n* = 14). At time of last data collection time point (May 7th, 2019), eight patients were still receiving HDT. After HDT initiation, serum testosterone levels rose from castrate levels to median nadir testosterone concentrations of 309 ng/dL (109–1134), 383 ng/dL (60–757), and 280 ng/dL (74–296) for HDT administrations of 2w, 3w, and 4w, respectively.

**Figure 1:**
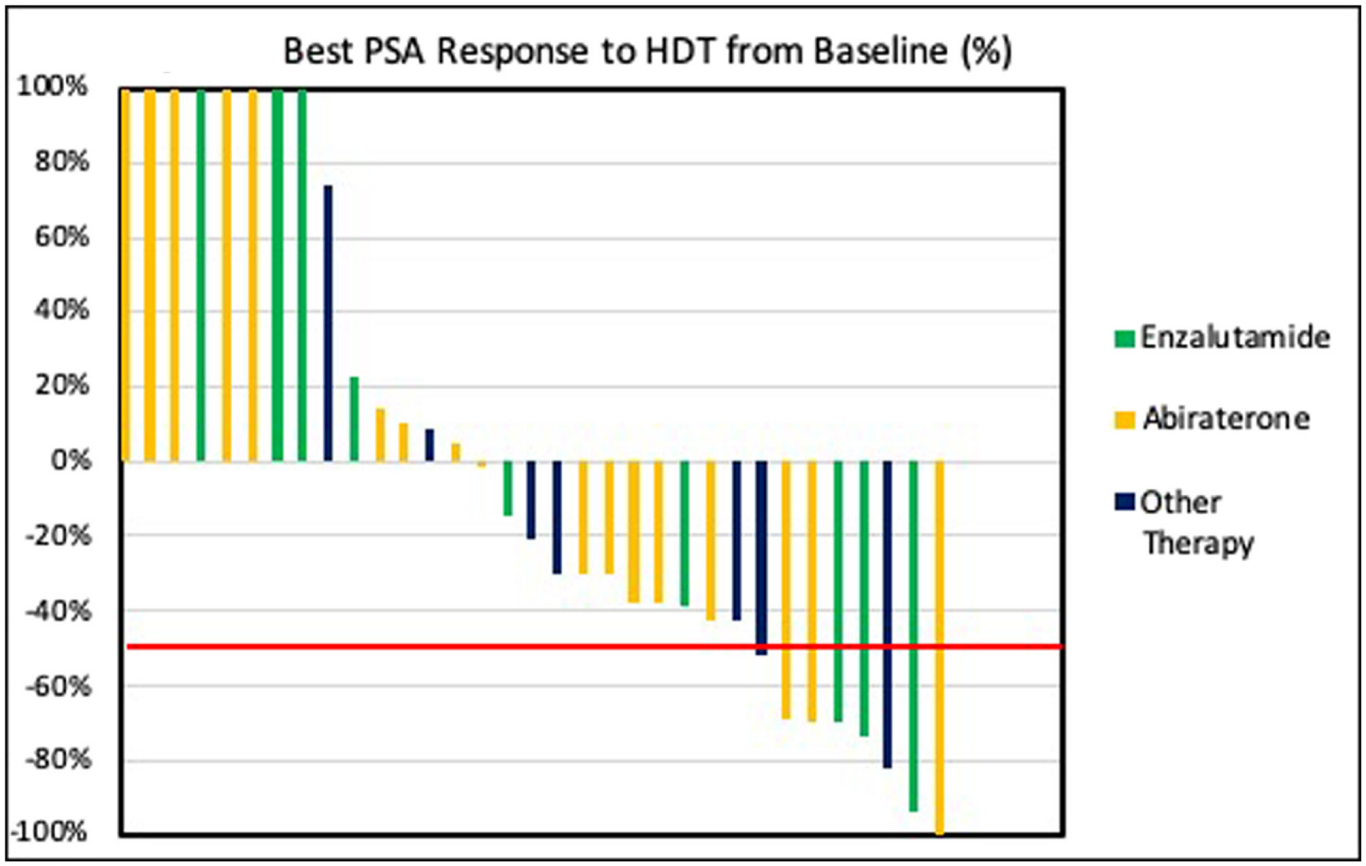
PSA waterfall plot of best PSA response in patients treated with HDT.

With median follow-up time of 5.9 months (1.8–27.6), HDT was administered for median of 5 (2–15) cycles and for median treatment duration of 4.0 months (95% CI, 2.6–5.3). Almost one fourth (24%) of patients achieved PSA50 and 48% of patients achieved PSA30 (Figure 1). Median duration of response for PSA50 responders was 2.3 months (95% CI, 1.7–2.8). Eight patients (24%) had an initial PSA rise with subsequent decline past their baseline PSA. Median time to PSA response (PSA50) was 0.9 months (95% CI, 0.3–1.4). Over three quarters of patients (79%, 26/33) achieved confirmed PSA progression leading to median TPP of 2.8 months (95% CI, 2.1–3.4). Patients with elevated baseline LDH levels (≥240 units/L) (*p* = 0.038) were associated with worse treatment outcomes.

After HDT discontinuation, twenty patients were re-challenged with abiraterone (*n* = 2) or enzalutamide (*n* = 18) with 30% (6/20) PSA50 responses and median duration of response of 1.2 months (0.7–3.7). Five patients received enzalutamide before and after HDT with 40% (2/5) achieving PSA50. Thirteen patients received abiraterone prior to HDT and subsequent enzalutamide following HDT discontinuation. Seven patients were still undergoing HDT at time of last data collection with 31% (4/13) PSA50 responses so far. Two patients received abiraterone before and after HDT with no PSA responses observed.

Overall, HDT administration was safe and tolerated well with few reports of new or worsening symptoms. Adverse events (AEs) of any cause or grade were reported in less than a third (30%, 10/33) of the cases and included insomnia (*n* = 1), acute pain (*n* = 3), rash (*n* = 1), urinary obstruction (*n* = 1), acne (*n* = 1), asthenia (*n* = 1), thrombocytopenia (*n* = 1), and gynecomastia (*n* = 1). Two grade 4 AEs involving asthenia and thrombocytopenia were observed during treatment with HDT and possibly related to HDT. No dose-limiting toxicities or treatment-related deaths were observed.

### Genomic findings

All patients received baseline ctDNA testing, with median time from testing to HDT initiation of 0.4 months (95% CI, 0.0–0.9). ctDNA testing revealed AR alterations in 39% of patients (amp, *n* = 7; mut, *n* = 5; both, *n* = 1); 48% TP53, and 12% DNA repair (ATM *n* = 1; BRCA2 *n* = 2; BRCA1 *n* = 1). Seven patients were positive for germline alterations (BRCA2 *n* = 2; BRCA1 *n* = 1; ATM *n* = 1; HOXB13 *n* = 1; PMS2 *n* = 1; MUTYH *n* = 2) with one patient having both BRCA2 and MUTYH.

No significant association was detected between baseline AR, TP53 and, DNA repair alterations with PSA response and TPP after HDT. With median baseline ctDNA% of 1.10 (0–80.9), patients were categorized above and below the median. Although no statistically significant difference was observed, patients above the median had numerically worse TPP outcomes (*p* = 0.435) (ctDNA% ≤1.10, 4.2 months (95% CI, 0.9–7.4); ctDNA% >1.10, 2.6 months (95% CI, 1.6–3.5).

With three patients still on HDT at time of last data collection, all patients with baseline AR alterations with repeated ctDNA data (69%, 9/13), achieved decreased expression of those AR alterations (Table 2). AR mutations receiving alteration knockdown were W742C, T878A, L702H, M896V, and V716M. PSA responses for this subset of patients were as follows PSA50 (*n* = 2), PSA30 (*n* = 3), and non-responders (*n* = 4). Only one patient receiving a repeated ctDNA assay reported a new detection of an AR alteration (AR W742L).

**Table 2:** HDT regulation of AR expression and response to antiandrogen post-HDT

Patient	AR Alteration	ctDNA%/PCN Pre-HDT	HDT Responder	ctDNA%/PCN Post-HDT	Abi or Enza Post-HDT	PSA50 Responder
1	Amplification	3.9	N	ND	Enza	Y
2	AR (W742C)	1.30%	PSA50	ND	Abi	N/A
3	Amplification	1.6	PSA30	ND	Abi	N
4	Amplification	1.5	PSA50	ND	Abi	N/A
5	AR (L702H)	34.90%	N	6.80%	Enza	N
	AR (V716M)	1.20%		ND		
6	Amplification	36.2	N	8.7	Enza	N/A
7	Amplification	1.4	N	ND	Enza	Y
	AR (W742L)	ND		1.20%		
8	AR (T878A)	0.80%	PSA30	ND	Enza	N/A
9	AR (W742C)	0.50%	PSA30	ND	Enza	N/A
	AR (M896V)	5.30%		ND		

DNA repair alterations, somatic and germline, were detected in 12% of patients (germline-only, *n* = 1; germline and somatic, *n* = 3). Somatic DNA repair alterations detected, included BRCA2 (C2363fs; W2970*; L2357fs), BRCA1 (M1?), and ATM (R3008C). Germline DNA repair alterations included BRCA2 (V1486Nfs; L2357Vfs), BRCA1 (Initiator Codon), and ATM (L762Vfs). One patient with DNA repair alterations achieved PSA50 while two achieved PSA30 responses. The BRCA1 germline and somatic patient achieved PSA50 after one cycle of HDT. One patient with germline HOXB13 (G84E) had an ongoing PSA50 response after five cycles of HDT and four months of therapy.

## DISCUSSION

The adaptive upregulation of AR within mCRPC is an important component of disease progression and resistance to effective androgen deprivation therapy and antiandrogens [5]. To our knowledge, this was one of the first clinical studies to suggest that HDT has the ability to alter AR adaptation potentially creating additional opportunities for further AR-signaling inhibition. In this study, HDT was safe and active in a subset of mCRPC patients and despite small numbers, those with DNA repair genes might yield higher responses.

In general, biochemical responses were observed in approximately one third of the patients with time to PSA progression slightly under three months, which was very similar to findings reported in the ongoing RESTORE trial which reported 30% PSA50 and median TPP of over 3 months in patients treated with BAT in their enzalutamide re-challenge cohort [16]. Whether PSA50 represent true disease response to re-challenge strategy or just a return to pre-HDT baseline levels is not completely clear.

Decreased AR expression experienced in all patients with baseline AR alterations was very interesting, but somewhat expected due to the elimination of the androgen-deprived state. Whether the low re-detection rate of the same AR alteration is a consequence of the lower burden of ctDNA in the blood post-treatment rather than an actual change in the AR status of the tumor, is unknown. Additionally, the impact of confounding factors such as glucocorticoid receptor upregulation in the mCRPC state should be considered. But it is at least plausible that HDT might have the potential to manipulate the adaptive nature of AR and a potential re-sensitization to further AR-signaling inhibition after HDT. To our knowledge, this has not been studied before in the clinical setting and requires further validation.

As suggested in previous studies, patients challenged to abiraterone post-HDT received limited success while responses to enzalutamide post-HDT were higher [16]. Whether enzalutamide’s inhibitory activity on the AR is a more adequate approach post-HDT than abiraterone is unknown. Interestingly, D4A, an abiraterone metabolite, has a comparable AR affinity to that of enzalutamide [17]. AR-V7 examination was not included in this study, but investigation into HDT’s effect on AR-V7 mutant patients should be of interest. Further investigation is required to assess the optimal sequence of oral antiandrogen administration before and after HDT.

Although no significant associations between baseline ctDNA and TPP were detected, the serologic responses observed in DNA repair patients was intriguing. Previous studies have demonstrated the AR acts as a licensing factor in DNA replication in androgen sensitive prostate cancer cells and that androgens induce double-strand DNA breaks within prostate cancer cell lines [18–21]. Potentially, tumors with DNA repair alterations are more sensitive to HDT. In a recent study, supraphysiologic androgen was shown to repress genes in DNA repair and delay restoration of damaged DNA and an enhanced effect in patients with DNA damage repair genes was observed [22]. Additionally, a case report on a germline BRCA2 and ATM positive patient treated with BAT achieved an undetectable PSA after 2 cycles, complete radiographic response after 6 cycles, and unmeasurable disease after 20 cycles [23]. With these experiences and eighty percent of our DNA repair patients achieving serologic responses, further examination into HDT exposure with DNA repair defects and in combination with DNA-repair targeted therapies is warranted.

Importantly, no major safety signals were identified. Regular intramuscular injections of testosterone cypionate was safe with no treatment discontinuation due to side effects, low number of significant AEs, and no treatment-related deaths. It is likely that our selected population with good performance status, low number of prior CRPC therapies, and bone-predominant disease have contributed to this favorable toxicity profile.

The main limitations of our study revolve around its retrospective nature, small patient size, limited access to metastatic lesion biopsies, heterogenous testosterone administration, and single center experience. Although patients received consistent forms of HDT, there was variation in the dosing interval and peak levels of testosterone were not measured in this real-world experience. Despite responses and time to progression were in line with published data, radiographic progression could not be assessed in this pilot, small study. Abiraterone and enzalutamide post-HDT data is premature and further analysis is necessary to confirm the efficacy of tumor re-senitization. Finally, while tissue biopsies remain the gold standard for detecting somatic alterations, ctDNA assays were used in this study. Known challenges of tissue biopsies of metastatic lesions include higher cost, location of metastatic sites, and potential biopsy failures [24]. Recently, Wyatt et al. examined the concordance of metastatic lesions and ctDNA demonstrating ctDNA assays sufficiently detect clinically relevant DNA driver alterations [24]. Concordance between ctDNA and tissue remains an important question and needs to be further evaluated.

In conclusion, in this retrospective study, exogenous testosterone for mCRPC led to a decrease in AR copy number and mutations which was independent from response to therapy. Further understanding of the genomic alterations predicting HDT response is required to explore the role of DNA repair and other genomic implications for optimal patient selection for this therapy.

## MATERIALS AND METHODS

This was a retrospective, real-world analysis of consecutive mCRPC patients treated with ≥2 testosterone cypionate injections at Tulane Cancer Center, New Orleans, LA between May 2016 and April 2019. Patients included were men ≥18 years with Eastern Cooperative Oncology Group (ECOG) performance status ≤2, confirmed prostate-specific antigen (PSA) progression with castrate serum testosterone levels (≤50 ng/dL), and documented metastases via CT scan and/or nuclear medicine bone scan. HDT was defined as administration of testosterone via intramuscular 400 mg testosterone cypionate injections q 2–4 w while receiving continuous luteinizing hormone-releasing hormone agonist therapy. Patients receiving any form of HDT as part of a clinical trial were excluded as well as those patients without NGS data in ctDNA.

Following PSA progression on HDT, patients could be re-challenged with an AR androgen inhibitor with either abiraterone or enzalutamide, per treating physician’s discretion. Patient clinical laboratory values, including PSA, hemoglobin, alkaline phosphatase, lactate dehydrogenase (LDH), and nadir testosterone, defined as the testosterone level at the clinic visit following HDT initiation, were monitored at baseline and each clinic visit following HDT initiation.

Prior to HDT initiation, NGS was performed (Guardant360, Redwood City, CA) to collect baseline circulating-tumor DNA (ctDNA) alterations including amplifications (amps) and mutations (muts; allele fraction ≥0.3%). Subsequent ctDNA testing following HDT progression was performed but was optional, per treating physician’s discretion. Guardant360 assay examines ctDNA detecting point mutations (73 genes), indels (23 genes), copy number amplifications (18 genes), and fusions (6 genes) in select genes and exons. All patients were consented for and received germline genetic testing (Invitae Corporation, San Francisco, CA) prior to HDT initiation. Examination of electronic medical records was performed to collect baseline clinical characteristics and assess clinical outcomes and adverse events. Clinical outcomes assessed included PSA response, time to PSA progression (TPP), and adverse events (AEs).

PSA responses (PSA50 and PSA30) were defined as PSA decline of ≥50% or ≥30% from baseline PSA level prior to HDT initiation. PSA progression was defined as 25% increase from nadir PSA (confirmed by a second rising PSA four weeks later) per Prostate Cancer Working Group 3 (PCWG3). Patients receiving an initial rise in PSA after receiving one dose of HDT continued therapy until a second confirmatory rising PSA. Time to PSA progression (TPP) was defined as the time from HDT initiation to PSA progression. Duration of response, defined as time from PSA50 to TPP, was assessed for PSA50 responders. Clinical outcomes in relation to abiraterone and enzalutamide re-challenge were assessed including treatment duration and PSA50. PSA50 with respect to abiraterone and enzalutamide re-challenge was defined as PSA decline of ≥50% from baseline PSA level post-HDT. Lines of CRPC therapy included new therapies or re-challenge with same therapy during the disease course (eg, a sequence of enzalutamide, HDT, enzalutamide was considered 3 lines of therapy).

Safety was assessed at each clinical visit (clinical visit before each testosterone administration for testosterone cypionate administration). Adverse events of any cause were collected consistently from data in clinical records.

Statistical analyses including the association of ctDNA and clinical characteristics with clinical outcomes, including PSA responses and TPP, were conducted. Chi-square test, fisher exact test, and Kaplan-Meier estimator were used and a *p*-value < 0.05 considered statistically significant.

## References

[1] de Bono JS , Logothetis CJ , Molina A , Fizazi K , North S , Chu L , Chi KN , Jones RJ , Goodman OB Jr , Saad F , Staffurth JN , Mainwaring P , Harland S , et al. Abiraterone and increased survival in metastatic prostate cancer. N Engl J Med. 2011; 364:1995–2005. 10.1056/NEJMoa1014618. 21612468 PMC3471149

[2] Scher HI , Fizazi K , Saad F , Taplin ME , Sternberg CN , Miller K , de Wit R , Mulders P , Chi KN , Shore ND , Armstrong AJ , Flaig TW , Flechon A , et al. Increased survival with enzalutamide in prostate cancer after chemotherapy. N Engl J Med. 2012; 367:1187–1197. 10.1056/NEJMoa1207506. 22894553

[3] Azad AA , Eigl BJ , Murray RN , Kollmannsberger C , Chi KN . Efficacy of enzalutamide following abiraterone acetate in chemotherapy-naive metastatic castration-resistant prostate cancer patients. Eur Urol. 2015; 67:23–29. 10.1016/j.eururo.2014.06.045. 25018038

[4] Robinson D , Van Allen EM , Wu YM , Schultz N , Lonigro RJ , Mosquera JM , Montgomery B , Taplin ME , Pritchard CC , Attard G , Beltran H , Abida W , Bradley RK , et al. Integrative clinical genomics of advanced prostate cancer. Cell. 2015; 161:1215–1228. 10.1016/j.cell.2015.05.001. 26000489 PMC4484602

[5] Linja MJ , Savinainen KJ , Saramaki OR , Tammela TL , Vessella RL , Visakorpi T . Amplification and overexpression of androgen receptor gene in hormone-refractory prostate cancer. Cancer Res. 2001; 61:3550–3555. 11325816

[6] Egan A , Dong Y , Zhang H , Qi Y , Balk SP , Sartor O . Castration-resistant prostate cancer: adaptive responses in the androgen axis. Cancer Treat Rev. 2014; 40:426–433. 10.1016/j.ctrv.2013.09.011. 24139549

[7] Antonarakis ES , Lu C , Wang H , Luber B , Nakazawa M , Roeser JC , Chen Y , Mohammad TA , Chen Y , Fedor HL , Lotan TL , Zheng Q , De Marzo AM , et al. AR-V7 and resistance to enzalutamide and abiraterone in prostate cancer. N Engl J Med. 2014; 371:1028–1038. 10.1056/NEJMoa1315815. 25184630 PMC4201502

[8] Azad AA , Volik SV , Wyatt AW , Haegert A , Le Bihan S , Bell RH , Anderson SA , McConeghy B , Shukin R , Bazov J , Youngren J , Paris P , Thomas G , et al. Androgen Receptor Gene Aberrations in Circulating Cell-Free DNA: Biomarkers of Therapeutic Resistance in Castration-Resistant Prostate Cancer. Clin Cancer Res. 2015; 21:2315–2324. 10.1158/1078-0432.CCR-14-2666. 25712683

[9] Annala M , Vandekerkhove G , Khalaf D , Taavitsainen S , Beja K , Warner EW , Sunderland K , Kollmannsberger C , Eigl BJ , Finch D , Oja CD , Vergidis J , Zulfiqar M , et al. Circulating Tumor DNA Genomics Correlate with Resistance to Abiraterone and Enzalutamide in Prostate Cancer. Cancer Discov. 2018; 8:444–457. 10.1158/2159-8290.CD-17-0937. 29367197

[10] Denmeade SR , Isaacs JT . Bipolar androgen therapy: the rationale for rapid cycling of supraphysiologic androgen/ablation in men with castration resistant prostate cancer. Prostate. 2010; 70:1600–1607. 10.1002/pros.21196. 20607766 PMC4124628

[11] Mohammad OS , Nyquist MD , Schweizer MT , Balk SP , Corey E , Plymate S , Nelson PS , Mostaghel EA . Supraphysiologic Testosterone Therapy in the Treatment of Prostate Cancer: Models, Mechanisms and Questions. Cancers (Basel). 2017; 9:166. 10.3390/cancers9120166. 29210989 PMC5742814

[12] Morris MJ , Huang D , Kelly WK , Slovin SF , Stephenson RD , Eicher C , Delacruz A , Curley T , Schwartz LH , Scher HI . Phase 1 trial of high-dose exogenous testosterone in patients with castration-resistant metastatic prostate cancer. Eur Urol. 2009; 56:237–244. 10.1016/j.eururo.2009.03.073. 19375217 PMC2738932

[13] Szmulewitz R , Mohile S , Posadas E , Kunnavakkam R , Karrison T , Manchen E , Stadler WM . A randomized phase 1 study of testosterone replacement for patients with low-risk castration-resistant prostate cancer. Eur Urol. 2009; 56:97–103. 10.1016/j.eururo.2009.02.022. 19282098 PMC2885777

[14] Schweizer MT , Antonarakis ES , Wang H , Ajiboye AS , Spitz A , Cao H , Luo J , Haffner MC , Yegnasubramanian S , Carducci MA , Eisenberger MA , Isaacs JT , Denmeade SR . Effect of bipolar androgen therapy for asymptomatic men with castration-resistant prostate cancer: results from a pilot clinical study. Sci Transl Med. 2015; 7:269ra2. 10.1126/scitranslmed.3010563. 25568070 PMC4507510

[15] Schweizer MT , Wang H , Luber B , Nadal R , Spitz A , Rosen DM , Cao H , Antonarakis ES , Eisenberger MA , Carducci MA , Paller C , Denmeade SR . Bipolar Androgen Therapy for Men With Androgen Ablation Naive Prostate Cancer: Results From the Phase II BATMAN Study. Prostate. 2016; 76:1218–1226. 10.1002/pros.23209. 27338150

[16] Teply BA , Wang H , Luber B , Sullivan R , Rifkind I , Bruns A , Spitz A , DeCarli M , Sinibaldi V , Pratz CF , Lu C , Silberstein JL , Luo J , et al. Bipolar androgen therapy in men with metastatic castration-resistant prostate cancer after progression on enzalutamide: an open-label, phase 2, multicohort study. Lancet Oncol. 2018; 19:76–86. 10.1016/S1470-2045(17)30906-3. 29248236 PMC5875180

[17] Li Z , Bishop AC , Alyamani M , Garcia JA , Dreicer R , Bunch D , Liu J , Upadhyay SK , Auchus RJ , Sharifi N . Conversion of abiraterone to D4A drives anti-tumour activity in prostate cancer. Nature. 2015; 523:347–351. 10.1038/nature14406. 26030522 PMC4506215

[18] Murthy S , Wu M , Bai VU , Hou Z , Menon M , Barrack ER , Kim SH , Reddy GP . Role of androgen receptor in progression of LNCaP prostate cancer cells from G1 to S phase. PLoS One. 2013; 8:e56692. 10.1371/journal.pone.0056692. 23437213 PMC3577675

[19] D’Antonio JM , Vander Griend DJ , Isaacs JT . DNA licensing as a novel androgen receptor mediated therapeutic target for prostate cancer. Endocr Relat Cancer. 2009; 16:325–332. 10.1677/ERC-08-0205. 19240183 PMC3072142

[20] Litvinov IV , Vander Griend DJ , Antony L , Dalrymple S , De Marzo AM , Drake CG , Isaacs JT . Androgen receptor as a licensing factor for DNA replication in androgen-sensitive prostate cancer cells. Proc Natl Acad Sci U S A. 2006; 103:15085–15090. 10.1073/pnas.0603057103. 17015840 PMC1622781

[21] Haffner MC , Aryee MJ , Toubaji A , Esopi DM , Albadine R , Gurel B , Isaacs WB , Bova GS , Liu W , Xu J , Meeker AK , Netto G , De Marzo AM , et al. Androgen-induced TOP2B-mediated double-strand breaks and prostate cancer gene rearrangements. Nat Genet. 2010; 42:668–75. 10.1038/ng.613. 20601956 PMC3157086

[22] Chatterjee P , Schweizer MT , Lucas JM , Coleman I , Nyquist MD , Frank SB , Tharakan R , Mostaghel E , Luo J , Pritchard CC , Lam HM , Corey E , Antonarakis ES , et al. Supraphysiological androgens suppress prostate cancer growth through androgen receptor-mediated DNA damage. J Clin Invest. 2019; 130:4245–4260. 10.1172/JCI127613. 31310591 PMC6763228

[23] Teply BA , Kachhap S , Eisenberger MA , Denmeade SR . Extreme Response to High-dose Testosterone in BRCA2- and ATM-mutated Prostate Cancer. Eur Urol. 2017; 71:499. 10.1016/j.eururo.2016.09.020. 27692705 PMC5808412

[24] Wyatt AW , Annala M , Aggarwal R , Beja K , Feng F , Youngren J , Foye A , Lloyd P , Nykter M , Beer TM , Alumkal JJ , Thomas GV , Reiter RE , et al. Concordance of Circulating Tumor DNA and Matched Metastatic Tissue Biopsy in Prostate Cancer. J Natl Cancer Inst. 2017; 109:djx118. 10.1093/jnci/djx118. 29206995 PMC6440274

